# Characterization of the complete chloroplast genome of Ulleung-do Island endemic*, Zabelia insularis* (Caprifoliaceae), in Korea

**DOI:** 10.1080/23802359.2019.1692730

**Published:** 2019-11-20

**Authors:** Woong Lee, Ji Young Yang, Seung-Chul Kim, Jae-Hong Pak

**Affiliations:** a Research Institute for Dok-do and Ulleung-do Island, Kyungpook National University, Daegu, Republic of Korea;; b Department of Biological Sciences, Sungkyunkwan University, Suwon, Republic of Korea;; c Research Institute for Dok-do and Ulleung-do Island and Department of Biology, School of Life Sciences, Kyungpook National University, Daegu, Republic of Korea

**Keywords:** chloroplast genome, Ulleung-do Island, *Zabelia insularis*, Linnaea clade sensu lato, Caprifoliaceae

## Abstract

The first complete chloroplast genome sequence of Korean insular endemic to Ulleung-do Island, *Zabelia insularis*, was reported in this study. The plastome size was 158,100 bp in total length, with one large single copy (90,529 bp), one small single copy (17,235 bp), and two inverted repeat (IR) regions (IR_a_ and IR_b_, each with 25,168 bp). The overall GC content was 38.3% and the genome contained 130 genes, including 83 protein-coding, 37 transfer RNA, and 8 ribosomal RNA genes. Phylogenetic analysis of 15 representative plastomes within the Caprifoliaceae suggests that *Z. insularis* is closely related to the species in the genus *Patrinia*.

*Zabelia* (Rehder) Makino (1948) belongs to one of the smaller lineages (ca. 30 species in five genera), known as the Linnaea clade sensu lato (s.l.), in the family Caprifoliaceae (Jacobs et al. 2010). Of five genera, *Abelia* R.Br. and *Zabelia* are the two most species rich genera with 15 and 10 species, respectively (Hara 1983). *Zabelia* is predominantly found in parts of the Middle East (Afghanistan and Turkestan) and North and East Asia and has always been assumed to be closely related or even congeneric with *Abelia* in East and Central Asia (Hara 1983). *Zabelia* was initially included in *Abelia* section *Zabelia* (Rehder 1911) but later generic rank recognition was supported based on palynological and anatomical autapomorphies, as well as basic chromosome number (Erdtman 1952; Hisauchi and Hara 1954; Verlaque 1983; Hara 1983; Ogata 1991). Molecular phylogenetic studies elucidating the position of *Zabelia* and the intergeneric relationships of the Linnaea clade s.l. are limited (Kim et al. 2001; Pyck 2001; Zhou and Qian 2003; Bell and Donoghue 2005). Nevertheless, recent studies suggested that monophyletic lineage *Zabelia* is not closely related to presumably polyphyletic *Abelia* (Jacobs et al. 2010; Wang et al. 2015). In Korea, four taxa, *Z. insularis*, *Z. biflora*, *Z. tyaihyonii*, and *Z. densipila*, are currently recognized (Kim 2007; Hong et al. 2012). *Zabelia insularis* is endemic to Ulleung-do Island with very small population size and its taxonomic treatment with *Z. biflora* or *Z. corea* is often controversial (Paik and Lee 1989; Sun and Stuessy 1998). We sequenced the complete plastome of *Z. insularis* and assessed its phylogenetic position within Caprifoliaceae.

Total DNA (Voucher specimen: 37°29′05″N 130°54′48″E, KNU-Lee171010135) was isolated using the DNeasy plant Mini Kit (Quiagen, Carlsbad, CA) and sequenced by the Illumina platform (Macrogen, Seoul, Korea). A total of 42,371,728 paired-end reads were obtained and assembled *de novo* with Velvet v. 1.2.10 using multiple *k*-mers (Zerbion and Birney 2008). The tRNAs were confirmed using tRNAsacn-SE (Lowe and Eddy 1997). The complete plastome length of *Z*. *insularis* (MH376309) was 158,100 bp, with one large single copy region (LSC; 90,529 bp), one small single copy region (SSC; 17,235 bp), and two inverted repeat regions (IR_a_ and IR_b_; 25,168 bp each). The overall GC content was 38.3% and the plastome contained 130 genes, including 83 protein-coding, 8 rRNA, and 37 tRNA genes. A total of 19 genes were duplicated in the IR regions, including 7 tRNA, 4 rRNA, and 8 protein-coding genes. The complete *ycf*1 gene was included in the IR at the SSC/IR_a_ junction, while the partial *ycf*1 gene became a pseudogene and located at IR_b_/SSC junction. The complete *rpl*23 gene was included in the IR at the LSC/IR_b_ junction, whereas the partial *rpl*23 gene became a pseudogene and located at IR_a_/LSC junction.

Fifteen representative species of Caprifoliaceae, including *Z. insularis*, were aligned using MAFFT v.7 (Katoh and Standley 2013) and phylogenetic analysis was conducted using IQ-TREE v.1.6.7 (Nguyen et al. 2015). The maximum-likelihood (ML) tree (Figure 1) showed that *Z*. *insularis* is closely related to genus *Patrinia*.

**Figure 1.**
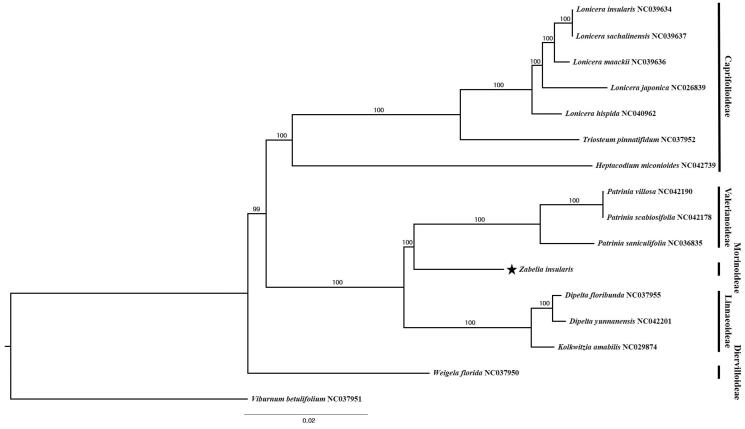
The ML tree based on 15 representatives of Caprifoliaceae and one outgroup taxon, *Viburnum betulifolium* (Adoxaceae). The bootstrap support value based on 1,000 replicates is shown on each node.
